# Molecular Epidemiology of Anthrax Cases Associated with Recreational Use of Animal Hides and Yarn in the United States

**DOI:** 10.1371/journal.pone.0028274

**Published:** 2011-12-09

**Authors:** Chung K. Marston, Christina A. Allen, Jodi Beaudry, Erin P. Price, Spenser R. Wolken, Talima Pearson, Paul Keim, Alex R. Hoffmaster

**Affiliations:** 1 Bacterial Special Pathogens Branch, Division of High-Consequence Pathogens and Pathology, National Center for Emerging and Zoonotic Infectious Diseases, Centers for Disease Control and Prevention, Atlanta, Georgia, United States of America; 2 Department of Biological Sciences, Northern Arizona University, Flagstaff, Arizona, United States of America; University of Iowa, United States of America

## Abstract

To determine potential links between the clinical isolate to animal products and their geographic origin, we genotyped (MLVA-8, MVLA-15, and canSNP analysis) 80 environmental and 12 clinical isolates and 2 clinical specimens from five cases of anthrax (California in 1976 [n = 1], New York in 2006 [n = 1], Connecticut in 2007 [n = 2], and New Hampshire in 2009[n = 1]) resulting from recreational handling of animal products. For the California case, four clinical isolates were identified as MLVA-8 genotype (GT) 76 and in the canSNP A.Br.Vollum lineage, which is consistent with the Pakistani origin of the yarn. Twenty eight of the California isolates were in the A.Br.Vollum canSNP lineage and one isolate was in the A.Br. 003/004 canSNP sub-group. All 52 isolates and both clinical specimens related to the New York and Connecticut cases were MLVA-8 GT 1. The animal products associated with the NY and CT cases were believed to originate from West Africa, but no isolates from this region are available to be genotyped for comparison. All isolates associated with the New Hampshire case were identical and had a new genotype (GT 149). Isolates from the NY, CT and NH cases diverge from the established canSNP phylogeny near the base of the A.Br.011/009. This report illustrates the power of the current genotyping methods and the dramatically different epidemiological conditions that can lead to infections (i.e., contamination by a single genotype versus widespread contamination of numerous genotypes). These cases illustrate the need to acquire and genotype global isolates so that accurate assignments can be made about isolate origins.

## Introduction

*Bacillus anthracis* is the etiologic agent of anthrax, a zoonotic disease which historically affected people in the United States who worked at textile mills or had other contact with contaminated animal products. In the USA, industrial processing of animal products accounted for the majority of human anthrax cases (n = 153) from 1955–1999 [1]. However, with improvements in industrial hygiene and the implementation of other practices (such as improved ventilation, vaccination of at-risk employees, decreased use of imported animal products), industrial cases of anthrax began to decline.

Reports of anthrax cases related to handling of animal products in a non-industrial setting are rare in the USA. In 1974, a cutaneous anthrax case was reported in a woman who bought drums made from goat hides in Haiti [2]. Prior to the 2001 bioterrorism-associated anthrax outbreak, the last fatal case of inhalation anthrax in the USA occurred in a self-employed, 32-year-old male home weaver in California in 1976 [3]. In follow-up investigations, the source of *B. anthracis* was determined to be contaminated hand-spun yarn consisting of various types of animal fibers (camel, goat, and sheep) imported from Pakistan [3], [4].

Recently, two inhalation and two cutaneous cases of anthrax were related to drum-making using imported goat hides. In 2006, two unrelated inhalation anthrax cases were reported in New York City and Scotland [1], [5]. In the New York City case, the patient, who recovered, made traditional African drums from processed hard-dried goat hides imported from West Africa. The case from Scotland also involved a drum-maker and was fatal. Additionally, in 2007, two cases of cutaneous anthrax were reported in a Connecticut drum-maker and his son [6]. This drum-maker also made West African drums using unprocessed hides. Lastly, in 2009, a gastrointestinal anthrax case was reported in a female drummer from New Hampshire [7]. In this case, the drummer did not make drums but attended a drumming event held in a community building where drums of various animal hides were played.

While *B. anthracis* has very little genetic variation, successful molecular subtyping methods have been developed within the last decade to examine phylogenetic relationships among isolates and as a molecular epidemiology tool to investigate anthrax cases. The original multiple-locus variable-number tandem repeats analysis system (MLVA) targeted eight loci (six chromosomally-located loci and one on each of the two virulence plasmids, pXO1 and pXO2) [8]. This method has been used to identify 89 *B. anthracis* genotypes (GTs), to study anthrax ecology, and to genotype isolates during the 2001 bioterrorism-associated anthrax outbreak. The MLVA-15 scheme includes seven additional loci and allows for increased differentiation of isolates [9]. Canonical single nucleotide polymorphism (canSNPs) analysis has been used to identify major clonal lineages within *B. anthracis* [9]. CanSNP analysis, in concert with MLVA-15, was used to subtype over 1000 isolates from 42 countries and subdivided the three major *B. anthracis* lineages into 12 clonal sub-lineages or sub-groups and 221 MLVA-15 genotypes [9].

In this report, we describe the molecular epidemiology of the anthrax cases linked to the recreational use of animal products occurring in the USA which includes the California and New York City inhalation anthrax cases, the two cutaneous cases from Connecticut, and the gastrointestinal case in New Hampshire. In all five cases, epidemiologic and environmental investigations were carried out to determine the source of exposure. For the 2006 and 2007 cases, law enforcement authorities were also involved to rule out any potential criminal activity. Numerous samples were collected from each patient's home, work space, or other associated facilities as part of the respective investigations. Culturing of the samples resulted in the recovery of many *B. anthracis* isolates. We utilized both MLVA-8 and MLVA-15, in addition to SNP analysis, to subtype both clinical and environmental isolates to determine 1) the genotype and canSNP lineage of the infectious isolate, 2) the genotypes of strains contaminating the patient's residence or work space, 3) the potential link between the source of the infectious isolate to animal products (goat hide, yarn, etc.) and 4) if the origin of the isolates as predicted by genotype is consistent with what is known about the origin of the animal products.

## Results

In this report, we analyzed 94 isolates and specimens by MLVA-8, MLVA-15 and canSNP analysis from five inhalation and cutaneous anthrax cases from a weaver (1976), three individuals associated with drum-making (2006, 2007), and a drummer (2009).

### 1976 California inhalation case

The six chromosomal loci of the MLVA-8 scheme were detected in all 29 isolates (Table 1). However, amplification of the pXO1 and/or the pXO2 loci failed for 19 (5 clinical, 14 environmental) of the isolates suggesting they lacked one or both virulence plasmids. This was further supported by additional plasmid specific PCR assays performed which also yielded negative results (data not shown). Seven isolates were missing one plasmid (three isolates cured of pXO1 and four isolates cured of pXO2) while the remaining 12 lacked both plasmids. Consequently, identification and assignment of a complete MLVA genotype (GT) was not possible for these isolates.

**Table 1:** MLVA-8 results of clinical and environmental *B. anthracis* isolates associated with the California, New York, Connecticut, and New Hampshire anthrax cases.

Isolate #	State	Source	pXO1	pXO2	CG3	*vrrB*_2_	*vrrB*_1_	*vrrA*	*vrrC*_1_	*vrrC*_2_	GT
**Clinical Isolates**											
4100	CA	Cerebrum	141	139	158	162	229	325	538	604	76
4099	CA	Cerebrum	141	139	158	162	229	325	538	604	76
4098	CA	Cerebrum	141	139	158	162	229	325	538	604	76
4286	CA	Pleural fluid	141	139	158	162	229	325	538	604	76
4281	CA	Pleural fluid	—	—	158	162	229	325	538	604	NA
4083	CA	Mediastinal node	—	—	158	162	229	325	538	604	NA
4085	CA	Mediastinal node	—	—	158	162	229	325	538	604	NA
4275	CA	CSF	—	—	158	162	229	325	538	604	NA
4274	CA	CSF	155*	—	158	162	229	313	538	604	NA
9149	NH	Blood	126	137	153	153	229	313	568*	613	149
0760	NY	Blood	123	137	153	162	229	313	604	613	1
**Clinical specimens**											
7851	CT	Biopsy	123	137	153	162	229	313	604	613	1
**Environmental isolates containing both plasmids**											
4277	CA	Yarn	132	139	158	162	229	313	538	604	71
4276	CA	Yarn	132	139	158	162	229	313	538	604	71
4246	CA	Loom	129	139	158	162	229	313	538	604	72
4255	CA	Yarn	129	139	158	162	229	313	538	604	72
4291	CA	Camel/Goat hair	126	143	158	153	229	313	538	604	105
4290	CA	Yarn	132	139	158	153	229	313	538	604	92
9147	NH	Drum	126	137	153	153	229	313	568*	613	149
1028	NY	Drum	123	137	153	162	229	313	604	613	1
4111	CT	Drum	123	137	153	162	229	313	604	613	1
**Environmental isolates cured of one or both virulence plasmids**											
4251	CA	Wool	—	—	158	153	229	313	538	604	NA
4096	CA	Yarn	—	—	158	153	229	313	538	604	NA
4297	CA	Camel/Goat hair	__	__	158	153	229	301	538	604	NA
4254	CA	Wool	—	—	158	153	229	301	538	604	NA
4280	CA	Wool	—	—	158	153	229	301	538	604	NA
4273	CA	Yarn	—	—	158	162	229	313	538	604	NA
4094	CA	Yarn	—	—	158	162	229	313	538	604	NA
4282	CA	Loom	—	—	158	162	229	313	538	604	NA
4090	CA	Yarn	132	—	158	153	229	289	538	604	NA
4093	CA	Yarn	132	—	158	162	229	313	538	604	NA
4259	CA	Yarn	120	—	158	162	229	313	400	532	NA
4091	CA	Yarn	—	139	158	162	229	313	538	604	NA
4095	CA	Yarn	—	139	158	153	229	301	538	604	NA
4285	CA	Human hair	—	139	158	153	229	301	538	604	NA

—, plasmid loci not detected.

NA, not applicable.

*, new allele size not previously described by Keim et al.

Among the nine clinical isolates, all four isolates containing both plasmids were identified as GT 76. An additional four isolates were cured of both plasmids but had identical allele sizes for all six chromosomal loci as the GT 76 isolates. However, one isolate, 4274, differed in allele size at the *vrrA* loci (313 vs. 325 bp) and also had an unusual pXO1 allele size (155 bp) which has not been previously documented. This isolate was in the A.Br.Vollum lineage by canSNP analysis.

**Figure 1:**
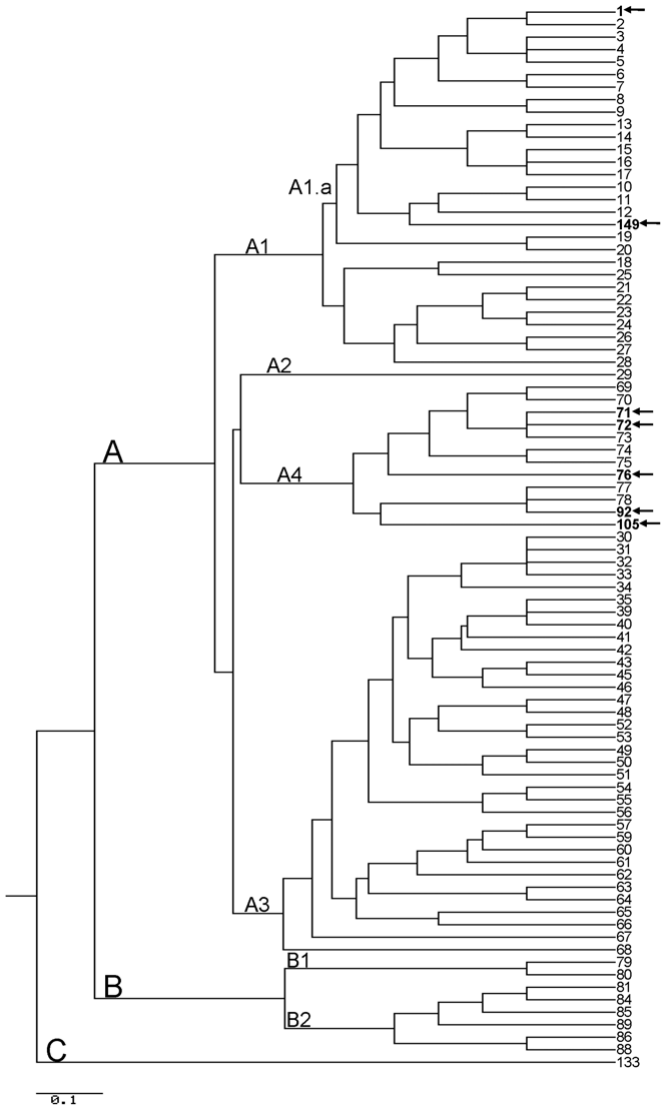
Dendrogram of MLVA-8 analysis of *B. anthracis* isolates collected from the 1976 California, 2006 New York, 2007 Connecticut anthrax cases, and 2009 New Hampshire case. All other genotypes are reference genotypes from Keim et al. [8]. For the California case, the clinical isolates were GT 76 (n = 4), while environmental isolates were GT 71 (n = 2), GT 72 (n = 2), GT 92 (n = 1), and GT 105 (n = 1). All clinical and environmental isolates from the New York (one clinical, 35 environmental) and Connecticut cases (one clinical specimen, 15 environmental isolates) were GT 1. All clinical (n = 2) and environmental (n = 9) isolates from the NH case were GT 149. Scale bar indicates amount of evolutionary change [12].

Of the environmental isolates containing both plasmids (n = 6), five isolates had MLVA-8 genotypes which had been previously described: two yarn isolates were GT 71 and one isolate from the loom and an additional yarn isolate were GT 72. In addition, one isolate (from yarn) was GT 92. One camel/goat hair isolate was GT 105 which has not been previously observed (Table 1, Figure 1).

**Figure 2:**
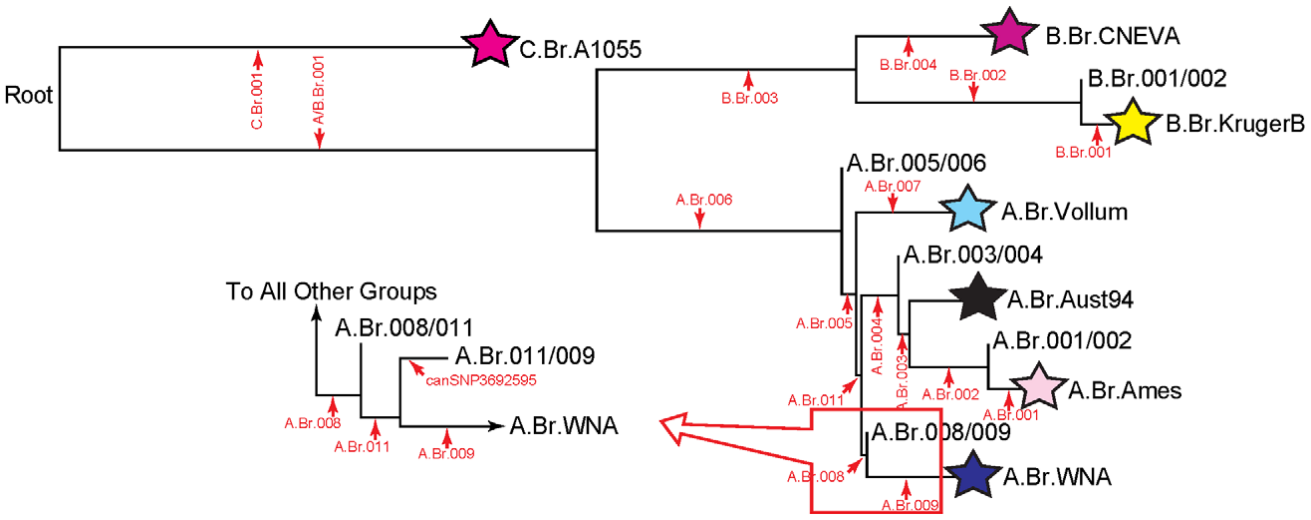
Phylogeny of the major groups of *B. anthracis* after Pearson et al. (2004) and VanErt et al. (2007). The new branch, “A.Br.011,” is flanked by branches A.Br.008 and A.Br.009. Thus, the group A.Br.008/009 is now subdivided into two groups: A.Br.008/011 and A.Br.011/009. The canSNP signature and assay that defines this new branch is provided in Table 3.

With one exception, all of the California isolates were in the A.Br.Vollum lineage by canSNP analysis. One isolate, 4259, was shown to be in the A.Br.003/004 sub-group (Table 2, Figure 2).

**Table 2:** Summary of molecular subtyping results for the California, New York, Connecticut, and New Hampshire anthrax cases.

Sample type	Number	MLVA-8 genotype(s)	Identical by MLVA-15	CanSNP lineage
**California case (1976)**				
Clinical isolates^a^	9	76 (n = 4)	NT	A.Br.Vollum (n = 9)
Environmental isolates^a^	20^c^	71 (n = 2), 72 (n = 2), 92 (n = 1), 105 (n = 1)	NT	A.Br.Vollum (n = 19), A.Br.003/004 (n = 1)^d^
**NY case (2006)**				
Clinical isolate	1	1	Yes	A.Br.011/009 (n = 1)
Environmental isolates	36	1	Yes	A.Br.011/009 (n = 36)
**CT cases (2007)**				
Clinical sample^b^	2	1 (n = 1)	Yes	NT
Environmental isolates	15	1	Yes	A.Br.011/009 (n = 15)
**NH case (2009)**				
Clinical isolate	2	149	Yes	A.Br.011/009 (n = 2)
Environmental isolates	9	149	Yes	A.Br.011/009 (n = 9)

NT, not tested.

a , Other MLVA-8 genotypes found but could not be assigned due to plasmid loss.

b , DNA from biopsy.

c ,14 isolates lacked one or both virulence plasmids and could not be genotyped.

d , canSNP lineage associated with one isolate, 4259.

### 2006 New York inhalation case

The clinical isolate from the patient was identified as MLVA-8 GT 1 (Table 2). Thirty-one environmental isolates collected from the patient's vehicle and work space were also identified as MLVA-8 GT 1. In addition, all 37 isolates were identical by MLVA-15. All isolates associated with this case were in the A.Br.011/009 sub-group (Table 2, Figure 2) by canSNP analysis.

### 2007 Connecticut cutaneous cases

While the biopsy specimen from the drum-maker did not amplify any of the MLVA loci, all MLVA-8 loci were amplified from the biopsy specimen from the drum-maker's son and identified as MLVA-8 GT 1. All environmental isolates from the drum-maker's home and shed where he processed the goat hides were also MLVA-8 GT 1 (Table 2). The clinical sample and the environmental isolates were identical by MLVA-15. All isolates associated with these two cases were in the A.Br.011/009 sub-group by canSNP analysis (Table 2).

### 2009 New Hampshire gastrointestinal case

The two clinical isolates from the patient and the nine environmental isolates recovered from the drums and the community building were identified as a new MLVA-8 genotype, designated GT 149 (Table 1). As in the CT and NY cases, the eleven isolates associated with this case were in the A.Br.011/009 sub-group by canSNP analysis (Table 2, Figure 2). Although the New Hampshire case isolate had a different MLVA-8 genotype than the New York and Connecticut cases, all isolates from these four cases were within the same canSNP lineage and clustered together by MLVA-8 (A1.a, Figure 1).

## Discussion

Among the five cases reported in this study, we were able to match clinical isolates or samples with environmental isolates in four of the cases (NY case, 2 CT cases, and NH case). Conversely, we were not able to match the clinical and environmental isolates associated with the California case. At the time of the California case (1976), molecular subtyping systems were not available to allow for more precise characterization of the clinical isolates and their possible linkage to environmental contamination. In this report, MLVA was used successfully to detect numerous genotypes in the environment of the California weaver's residence. Although none of the environmental isolate genotypes matched the clinical isolate genotypes, the clinical and environmental isolates that were completely genotyped appear to be closely related within the A4 MLVA cluster (Vollum cluster) as described by Keim et al. (Figure 1) [8]. Among the clinical isolates, all isolates were in the A.Br.Vollum canSNP lineage, and four out of nine clinical isolates were GT 76 and an additional four isolates had chromosomal allele sizes that were consistent with this genotype which has been described from Pakistan [8]. Thus, the MLVA-8 and canSNP analysis was consistent with the geographic origin of the yarn used by the weaver.

Based on the number of genotypes found in the environment of the California weaver's home, a co-infection of the patient might be expected. While all the clinical isolates were in the same canSNP lineage, we identified two MLVA-8 genotypes. Four of the isolates were GT 76, and one of the isolates, 4274, differed from the other clinical isolates at two loci demonstrating this isolate differs genotypically from GT 76. In addition, in the original investigation, two different biotypes of *B. anthracis* were observed [4]. One of the clinical isolates was poorly encapsulated and had low virulence for guinea pigs. This strain appeared to be non-encapsulated in colonial form on bicarbonate agar, but capsules were observed by immunofluorescence microscopy using anti-*B. anthracis* anti-serum [4]. Suffin et al. also report that these two same biotypes were seen in the yarn obtained in the weaver's home [4]. Unfortunately, we were not able to correlate these two biotypes with the MLVA genotypes identified in the clinical isolates due to plasmid loss. The presence of multiple genotypes may have resulted from the weaver's use of animal products originating from multiple sources (camel, goat, and sheep). In addition, the number of contamination events that occurred prior to the weaver's illness remains unknown.

None of the environmental isolates from the California case were an exact match to either clinical isolate genotype. However, several of the environmental isolates (GT 71 and 72) differed from the clinical isolates only at the pXO1 loci. Thus, the matching genotype among the environmental isolates may not have been identified due to the high rate of pXO1 loss in our archived isolates associated with this case. The isolates included in this report from the California case were stored for over 25 years at room temperature on agar slants with mineral oil overlay. We have previously documented that the storage conditions used for these isolates may have had an adverse effect on their plasmid stability and, thus, caused the loss of plasmids over the course of their long-term storage [10]. The loss of plasmids prevented assignment of complete MLVA-8 genotypes in some cases.

In contrast to the California case where multiple MLVA genotypes were found, the isolates collected in the NY, CT and NH cases were MLVA-8 GT 1 (NY and CT) and MLVA-8 GT 149 (NH), and all isolates from these three cases belong in the A.Br.011/009 lineage. The animal products associated with the NY and CT cases were believed to originate from West Africa, but only isolates from other African regions were available to be genotyped for comparison. The link between the canSNP lineage and the West African origin of the hides is surprising as only one isolate originating from Africa (isolated in Ethiopia) has been discovered to belong in this canSNP A.Br.011/009 lineage but possesses a different MLVA genotype. While molecular subtyping data exists for isolates from the southern parts of Africa, very little information is known regarding the molecular subtypes of isolates in the western region of Africa. Molecular subtyping of additional *B. anthracis* isolates from this region in Africa is necessary in order to gain a better understanding of the diversity of isolates in this area of the world.

While little is known regarding the diversity of *B. anthracis* strains in West Africa, outbreaks of anthrax continue to occur there [11]. Thus, animal products obtained from this region could be contaminated with *B. anthracis* spores. Public health agencies continue to recommend using only animal products which are free of *B. anthracis* spores (i.e., not using hides of unknown origin or from regions with epizootic anthrax such as West Africa) in order to reduce the potential for an anthrax infection [6]. In addition to the hides being contaminated with *B. anthracis* spores, the practice of drum-making, which creates aerosols, also contributed to the infections of both drum-makers and the CT drum-maker's child. The drum-making process involves scraping, stretching, and sanding the goat hides which likely produced aerosols contributing to the infections of the NY and CT cases.

Similar to the NY and CT cases, all of the isolates associated with the NH case were identical and MLVA-8 was used to successfully match the clinical and environmental isolates from this case. However, the NH isolates were a new genotype, GT 149. While this genotype differed from GT 1 at three loci (vrrB2, vrrC1, pXO1), it was within the same cluster (A1.a) as GT 1 (Figure 1) [8]. Indeed, canSNP analyses confirmed that these isolates are from the same lineage and the different MLVA genotypes suggest that further SNP analyses would provide additional resolution. All CT, NY, and NH isolates showed the ancestral state for canSNP3692595 (discovered by comparing the A0343 genome to other existing genomes), suggesting that the bifurcation point leading to these isolates lies near the base of the A.Br.011/009 lineage terminating in A0343 (Figure 2). While the origins of the drums used at the drumming event are not definitively known and, thus, a geographic link cannot be made, the isolates in the NH case are closely related to the isolates from the NY and CT cases.

In the current report, we used MLVA and canSNP analysis of *B. anthracis* to attempt to link clinical isolates with environmental isolates recovered from various locations associated with animal products used by the patients. MLVA and canSNP analysis successfully linked clinical and environmental isolates recovered in the NY, CT, and NH cases. Although none of the environmental isolate genotypes matched the clinical isolate genotypes in the CA case, the clinical and environmental isolates that were completely genotyped appear to be closely related (within the same MLVA-8 cluster) and genotypes identified from the yarn used by weaver were consistent with the yarn's geographic origin. These examples illustrate the power of the currently available genotyping methods and the dramatically different conditions that can be identified (i.e., contamination by a single event or genotype versus widespread contamination of numerous genotypes). In addition, the NY, CT, and NH cases illustrate the continued need to acquire and molecularly subtype isolates from around the world so that accurate predictions can be made about isolate origins.

## Methods

### Ethics statement

This report does not meet the definition of research under 45 CFR 46.102(d), the U. S. Code of Federal Regulations regarding the protection of human subjects. Thus, review from our institutional review board was not required, and a waiver was obtained. Specimens were initially obtained during the course of outbreak investigations and submitted for diagnostic testing; specific informed consent was not obtained, although individuals were free to decline. Activities that do not meet the definition of research are not subject to informed consent requirements under 45 CFR 46.

### Bacterial isolates

Twenty nine isolates related to the California inhalation case were included in this report (Table 1). Nine clinical isolates from the patient and 20 additional isolates from samples labeled as yarn, wool, camel/goat hair, human hair, or the loom used by the patient were assayed.

The clinical isolate from the patient and 36 environmental isolates were included from the 2006 New York City case. The environmental isolates were recovered from samples collected in the drum-maker's warehouse and home, from goat hides in the drum-maker's possession, and in the vehicle that was used to transport the goat hides.

No clinical isolates were recovered from the 2007 cutaneous anthrax cases from Connecticut. However, biopsy specimens from both patients were tested. In addition, 15 environmental isolates recovered from samples taken in the drum-maker's residence and work shed, previously made drums, vehicle and goat hides found in the work shed were included in this report.

Eleven isolates associated with the 2009 New Hampshire case were included in the report. Two clinical isolates from the patient and nine environmental isolates that were recovered from drums and the community building where the patient attended the drumming event were assayed.

Isolates were stored in 25% glycerol with water and held at −70°C until needed. The clinical specimens were stored at −20°C until processed. Prior to their inclusion in this study, the California isolates were stored at ambient temperature on tryptic soy agar slants overlayed with mineral oil in various physical locations for over 25 years until they were recovered from the slants in 2002 [10].

### DNA extraction

DNA template from all recovered isolates was obtained by heat-lysis of a single colony after overnight growth on SBA. Using a 1 µl loop, one colony was suspended in 200 µl of 10 mM Tris-HCl pH 8.0 in a 1.5-ml tube containing a 0.22 µM filter unit (Millipore, Billerica, MA). The suspension was heated at 95°C for 20 min and centrifuged in a microfuge at 6000× g for 2 min. The filter unit was discarded, and the cell lysate was held at −20°C until testing. One µl of lysate was used in each reaction.

DNA from clinical specimens was extracted using the QIAamp DNA Mini kit (Qiagen, Valencia, CA) according to manufacturer's instructions.

### MLVA

MLVA-8 and the expanded MLVA-15 were performed as previously described by Keim et al. and Van Ert et al., respectively [8], [9]. Briefly, six chromosomal (*vrrA*, *vrrB*_1_, v*rrB*_2_, *vrrC*_1_, *vrrC*_2_, CG3) and 2 plasmid (pXO1-aat, pXO2-at) loci were amplified for the MLVA-8 scheme. Seven additional loci (VNTR 12, VNTR16, VNTR17, VNTR 19, VNTR 23, VNTR 32, and VNTR 35) were amplified for the MLVA-15 scheme. Each amplicon was labeled with one of three dyes (FAM, HEX, or NED) and analyzed on an ABI 377 and/or ABI 3130 automated DNA sequencers (Applied Biosystems, Foster City, CA). ABI Genescan (ABI 377) and Genemapper v3.7 (ABI 3130) software (Applied Biosystems) were used to analyze gel images and fragment sizes, respectively.

### CanSNP analysis

CanSNP analysis was performed as previously described [9]. This technique categorizes isolates into one of 12 sub-lineages (C.Br.A1055, B.Br.CNEVA, B.Br.KrugerB, A.Br.Vollum, A.Br.Aust94, A.Br.Ames, A.Br.WNA,B.Br.001/002, A.Br.005/006, A.Br.003/004, A.Br.001/002, A.Br.008/009). During the course of this work, we identified an additional canSNP that provides resolution within the A.Br.008/009 group (Table 3). In keeping with the nomenclature system previously reported [9], this canSNP is called A.Br.011 and the two flanking sub-groups are A.Br.008/011 and A.Br.011/009. We also tested a canSNP (3692595; Table 3) located near the base of A.Br.011/009 to gain further resolution of our ABr011/009 isolates.

**Table 3:** Novel *B. anthracis* canSNP assays developed in this report.

Assay name	A.Br.011	A.Br.011/009_3692595
Assay target	Provides resolution within A.Br.008/009 group	SNP on A.Br.011/009 branch (terminal genome is A0343)
SNP Position (bp)^a^	2552486	3692595
Ancestral primer (5′-3′)^b^	AAACGAATTCCCGCTGAAAATAcTG	CCCTAAAAAAGCAGAGACTATgG
Derived primer (5′-3′)^b^	**CGGGGCGGGGCGGGGCGGGCG**AAACGAATTCCCGCTGAAAATAtTA	CCCTAAAAAAGCAGAGACTATcA
Consensus primer (5′-3′)	GATAAAAATCGGAATTGAAGCAGGA	CGCACATGAAGTGGAAGAAAGTACG
Assay format^c^	meltMAMA	SYBR MAMA

a Using ‘Ames ancestor’ genome (GenBank ref: AE017334).

b Underlined nucleotides indicate the position of the SNP; bolded nucleotides indicate an introduced GC clamp that increases the melt temperature of the primer, thus enhancing allelic discrimination [13]; small-case nucleotides represent deliberate mismatches incorporated into the allele-specific primers.

c All assays were optimized on an Applied Biosystems ABI PRISM 7900HT Sequence Detection System using default thermocycling parameters, with the addition of the dissociation curve [13], [14].
